# A Novel Three-Dimensional Analysis of Tongue Movement During Water and Saliva Deglutition: A Preliminary Study on Swallowing Patterns

**DOI:** 10.1007/s00455-018-9953-0

**Published:** 2018-10-31

**Authors:** Giannina Álvarez, Fernando José Dias, María Florencia Lezcano, Alain Arias, Ramón Fuentes

**Affiliations:** 1 Master’s Program in Dentistry, Dental School, Universidad de La Frontera, Temuco, Chile; 2 Department of Integral Adults Dentistry, Research Centre for Dental Sciences (CICO), Dental School, Universidad de La Frontera, Temuco, Chile; 3 Universidad Adventista de Chile, Chillán, Chile

**Keywords:** Deglutition, Deglutition disorders, Lingual movements, Electromagnetic articulography, Oral physiology, Bolus volume

## Abstract

Deglutition is a complex oral function, and the study of the whole process requires a precise analysis of the elements involved, especially of the tongue biomechanics. We described a three-dimensional analysis of tongue movements during both saliva and water deglutition in participants with normal occlusion. Fourteen participants (25.36 ± 4.85 years) were evaluated, and the movements of anterior, middle, and posterior portions of the tongue were recorded using AG501 3D-electromagnetic articulograph. An average volume (AVS) for water deglutition was determined for each participant. Saliva deglutition was classified according to Bourdiol et al. 35.71% was type I, 14.29% type II, and 50% type III. The greatest displacement on the inferior–superior axis was in the posterior portion, followed by the middle and anterior portions. In the posterior–anterior axis, smallest movement was in the anterior portion. During water deglutition, on the inferior–superior axis, there were statistical differences for 1-AVS between the anterior/middle and anterior/posterior portions of the tongue. There were statistical differences for both ½-AVS and ¼-AVS between the anterior/posterior portions of the tongue. On the posterior–anterior axis, there were no statistical differences among any volume–portion relations. On the medial–lateral axis, there was statistical difference for the ½-AVS between middle and posterior portions. The movement of the tongue portions was influenced by the volume and the element to be swallowed. The amplitude of the movement was directly proportional to the volume of water swallowed.

## Introduction

Deglutition is a complex biomechanical process purpose of which is to transport food and fluids from mouth to stomach through pharynx and esophagus [1–3]. This process requires the participation of neuronal systems involved in the integration of both sensitive stimuli and motor response [4, 5]. Any condition affecting this integration can lead to dysphagia [4]. The process of swallowing is sequential, coordinated, symmetric, semiautomatic, unique, and specific to each individual [6].

During swallowing and other oral functions such as chewing, the tongue contributes to the formation, placement, and propulsion of both fluid and solid bolus through the oropharynx [7]. The process of propulsion is determined by a coordinated elevation of the anterior, middle, and posterior portions of the tongue. This neuromuscular coordination is still under study [8]. The patterns of tongue movement during swallowing have been recorded and studied using different techniques and methodologies. Doods et al. [9] described two patterns of movement, *dipper* and *tipper,* employing videofluoroscopy. In the *dipper* pattern, the tongue apex submerges and lifts the fluid bolus from a sublingual position to a supralingual position reaching the palatal face of the upper incisors, whereas in the tipper pattern, the tip of the tongue and the fluid bolus are already in a posterior position. In both patterns, the movement of the tongue continues with undulatory, peristaltic, and elevatory movements of the two anterior thirds of the tongue making contact with the palate and pushing the fluid toward the posterior tongue portion and then toward the oropharynx. Bourdiol et al. [10], using 2D-electromagnetic articulograph, described three patterns of salivary swallowing: *Type I*, analogous to the tipper pattern; *Type II*, analogous to the dipper pattern; and *Type III*, in which the three portions of the tongue descend, the saliva is confined between the back of the tongue and the palate and, in a simultaneous upward movement of the three portions, the saliva is transported toward the oropharynx.

There are several techniques and methodologies to evaluate swallowing; however, the capability to analyze the complex movements associated to swallowing in a three-dimensional way is very limited [3, 11]. Among the most discussed techniques, we count videofluoroscopy, endoscopy, computed tomography, ultrasound, electromagnetic articulography, electromyography, manometry, and electropalatography [11]. Videofluoroscopy is considered the gold standard to study swallowing and consists of an X-ray video recording of oropharyngeal structures and bolus by adding a radiopaque contrast medium [12, 13]. The principal limitations of this technique are low temporal resolution, exposure to ionizing radiation [2, 14], and two-dimensional visualization and limited observation of tongue movements [3]. Another technique that has been used is electromagnetic articulography (EMA), which is based on the principles of electromagnetic induction to record the position of moving structures [15]. As a technique for the study of swallowing, it offers advantages over other procedures, both in the ability to collect data without biological risk with high precision (0.3 mm) and high temporal resolution (sample frequency of 1 kH) [11].

The importance of swallowing as a voluntary and involuntary process is frequently underrated but any alteration of this process can affect the stomatognathic system, its function, and the quality of life. It is necessary to contribute to the physiological findings and general knowledge in this area. The aim of this study is to analyze the lingual movement in a three-dimensional way, while swallowing either saliva or different volumes of water in adult participants, healthy and with normal occlusion. This analysis will be conducted in order to determine differences in the displacement of three different portions of the tongue (anterior, middle, and posterior) comparing its range of movement in the three directions of space. An important aspect of this study lays in the three-dimensional analysis of the tongue movement since previous studies were conducted principally analyzing only inferior–superior axis [9–11].

## Materials and Methods

### Sample

This descriptive study was carried out with 14 participants (seven men and seven women) older than 18 years of age, healthy, with normal occlusion, and continuous dental arch. The EAT-10 questionnaire, Eating Assessment Tool [16], was used to exclude participants with dysphagia problems. In addition, a clinical examination was performed to exclude participants with altered stomatognathic functions (swallowing, phonation, and/or breathing) and to register the characteristics of dental arches, intermaxillary relationships, tongue, and palate. The participants were referred to the Oral Physiology Laboratory of the Research Centre for Dental Sciences (Universidad de La Frontera, Temuco, Chile), where they received all the information of the investigation and signed an informed consent. This study was approved by the Scientific Ethics Committee of the Universidad de La Frontera (Approval Number 001/2017).

### Electromagnetic Articulography

**Fig. 1:**
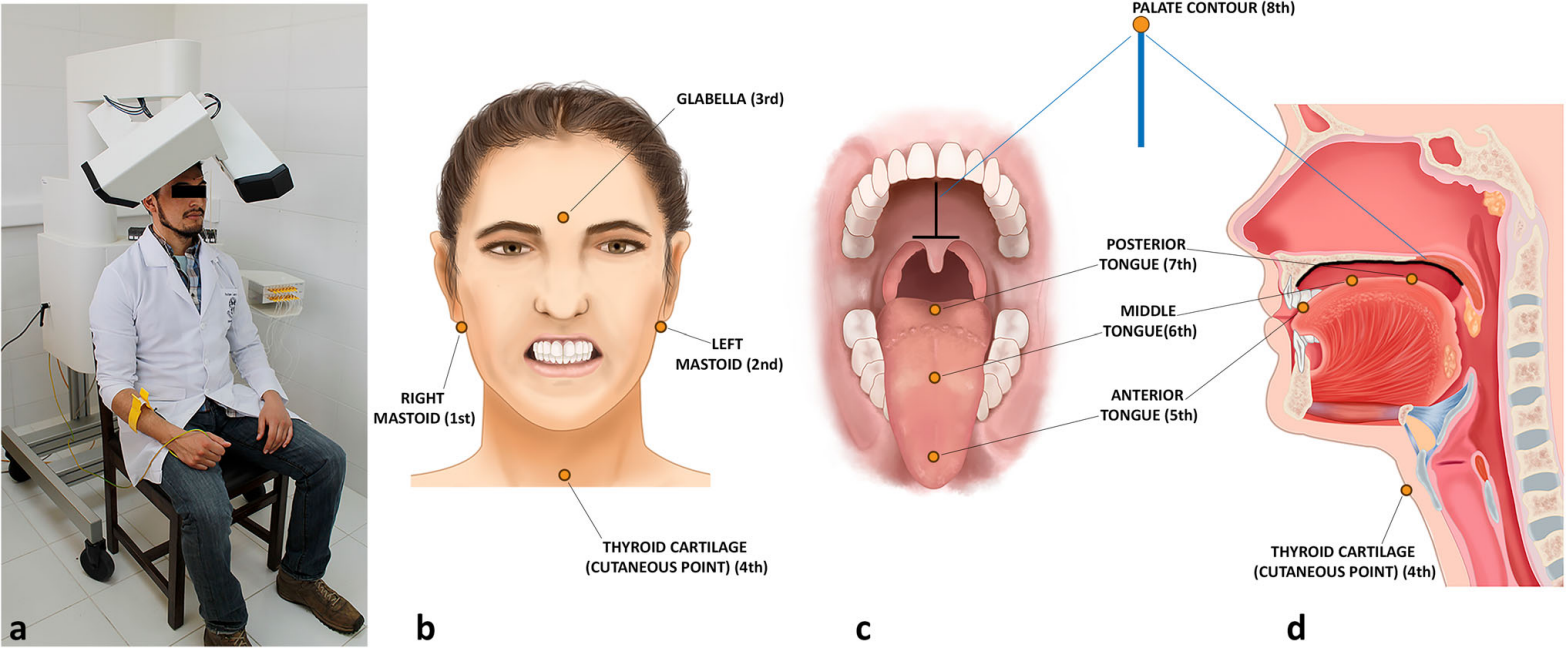
3D-EMA AG501 (Carstens Medizinelektronik) and sensors distribution diagram. **a** Participant in a recording session with 3D-EMA AG501. The sensors placed in the head of each participant must be positioned under the transmitter coils of the articulograph within the measurement area. **b** Frontal view of 3D-EMA extra-oral sensors (glabella, right and left mastoid, cutaneous point of thyroid cartilage). **c** Frontal view of 3D-EMA intra-oral sensors (anterior, middle, and posterior portions of the tongue; palate contour). **d** Sagittal view of 3D-EMA sensors

A 3D Electromagnetic Articulograph (AG501, Carstens Medizinelektronik, Bovenden, Germany; 3D-EMA; Fig. 1a) was employed to record tongue movement during swallowing on inferior–superior, posterior–anterior and medial–lateral axes.

Seven sensors were placed in the participant’s head at specific points as shown in Fig. 1b–d. The first three sensors were placed as reference to normalize the coordinates of the remaining sensors applying a specific function of the EMA AG501 called “Head Correction”. This allows the visualization and recording of the movement of interest for this study removing the movement of the participant’s head, as we previously reported for mandibular movement analysis [17].

The anterior sensor of the tongue was positioned 10 mm from the lingual apex; the posterior sensor was positioned in the most posterior, accessible and tolerable area according to Steele et al. [11]; and the middle sensor was placed between the first two sensors, equidistantly. The sensor placed at the cutaneous point of the thyroid cartilage was placed to identify the act of swallowing—principally during spontaneous swallowing of saliva—since thyroid cartilage describes an upward movement during this act. In addition, an extra sensor (eighth) attached to a wooden rod was used to delimitate the palate contour in the sagittal plane (axes z-inferior–superior and x-posterior–anterior), which served as an anatomic reference to analyze tongue movements. The palate contour was recorded from the base of the uvula to the retro-inter-incisive papilla (Fig. 1d).

### Recording Protocol

Each participant was previously instructed on the tasks to be performed during the recordings with 3D-EMA AG501. The participant was seated under the transmitter coils of the articulograph, in a comfortable and upright position. A few minutes were allowed to each participant to habituate him- or herself to the intra-oral sensors.

### Spontaneous Deglutition of Saliva

Tongue movement was recorded during spontaneous swallowing of saliva. Each participant was instructed to maintain a comfortable and upright position while watching a video displayed in a screen placed at 1 m from the participant. The participants were asked to relax and focus on the video with their mouth closed while the 3D-EMA operator performed three consecutive recordings of 1 min length each. The participants were not informed of the exact moment in which the recording was done in order to obtain recordings of spontaneous, involuntary, and noninduced swallowing of saliva.

#### Water Deglutition

Tongue movement was recorded while swallowing different volumes of water. An average volume of swallowing (AVS) was determined for each participant before placement of the EMA sensors. To determine the AVS, each participant was asked to drink in a single swallow a convenient and comfortable volume of water from a graduated beaker in order to register the volume swallowed. This procedure was repeated five times to calculate the mean volume swallowed or AVS.

The participants were asked to swallow 1-AVS, ½-AVS, and ¼-AVS. Each recording initiated when the participant was instructed to drink the water contained in the beaker (either 1-AVS, ½-AVS or ¼-AVS) and ended at the end of the oropharyngeal phase of swallowing, when the sensor glued to the cutaneous point of the thyroid cartilage indicated the end of the thyroid cartilage activity. Three repetitions of the task were recorded in order to obtain mean values of the parameters derived from the recordings.

### Data Processing

**Fig. 2:**
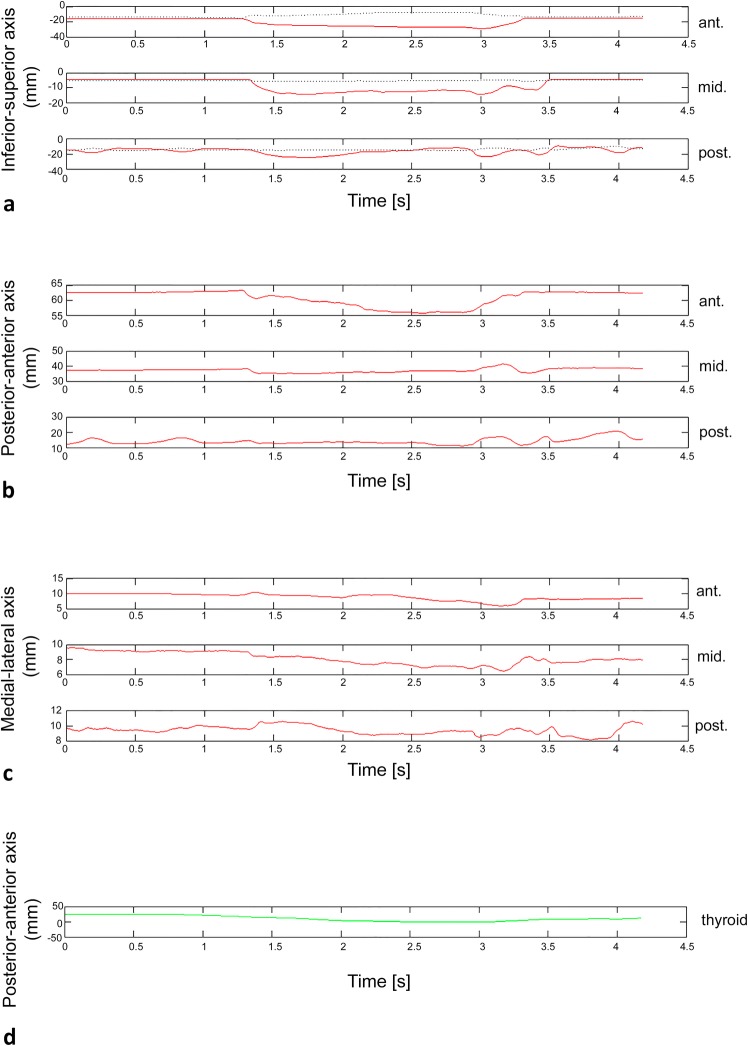
Graph of displacement (mm) versus time(s) for a single deglutition of 1-AVS of water. **a** Displacement of the three lingual portions on the inferior–superior axis. **b** Displacement of the three lingual portions on the posterior–anterior axis. **c** Displacement of the three lingual portions on the medial–lateral axis. **d** Displacement of the cutaneous point of thyroid cartilage on the posterior–anterior axis

All EMA AG501 data were stored in binary files and processed using MATLAB^®^ (MathWorks Inc., USA) in order to obtain intelligible data representation (Fig. 2) and to determine the range of motion of each tongue portion in the three anatomic axes (inferior–superior, medial–lateral, posterior–anterior).

### Swallowing Pattern Determination

The deglutition pattern type was determined according to Bourdiol classification (types I, II and III) [10]. To classify the deglutition pattern of each participant, graphs of inferior–superior position against time were analyzed. The pattern type was determined by the coordination of the tongue segments during upward and downward movements of the tongue when swallowing saliva. It is important to mention that all graphs were synchronized and shared the same time scale.

### Statistical Analysis

SigmaPlot 12.0 (Systat Software, Inc, San Jose, CA, USA) was used to perform the statistical analysis. The Shapiro–Wilk test was applied to determine the normality of the data. For data with normal distribution, one-way ANOVA (*α* = 0.05) was applied, followed by the Bonferroni post hoc test. Normal data were presented as mean ± standard deviation. For data whose distribution was not normal, the Kruskal–Wallis test and Dunn’s post hoc test were applied; this data was presented as median, low (25%) and high (75%).

## Results

### General Features

The mean age of the participants was 25.36 ± 4.85 years (women 25.38 ± 5.04 years; men 26.18 ± 5.15 years), and the mean of AVS of the participants was 20.63 ± 9.29 ml (women 21.25 ± 9.29 ml; men 19.91 ± 9.87 ml). There were no statistical differences between different AVS by sex (*p* = 0.948).

### Swallowing Patterns

Regarding identification of tongue movement patterns according to those described by Bourdiol et al. [10], it was observed that 35.71% (*n* = 5) of our participants were type I, 14.29% (*n* = 2) were type II, and 50% (*n* = 7) were type III.

### Tongue Movement During Spontaneous Deglutition of Saliva

**Fig. 3:**
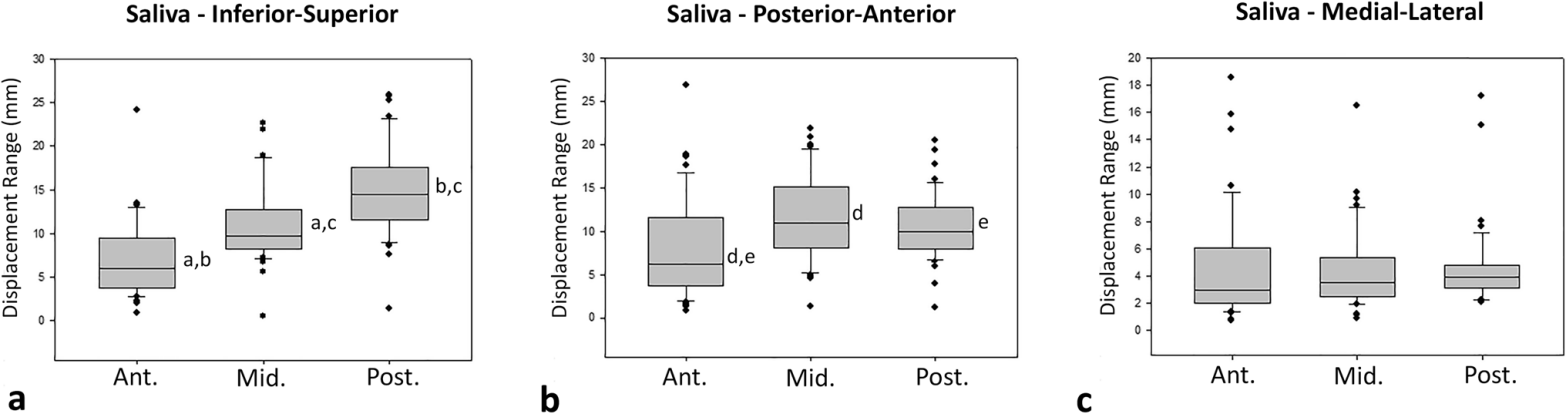
Range of displacement of the anterior, middle, and posterior portions of the tongue during spontaneous deglutition of saliva on the **a** inferior–superior, **b** posterior–anterior, and **c** medial–lateral axes. The letters (a–e) indicate intra-graph significant differences between different tongue portions

Table 1 shows the displacement range in millimeters of the anterior, middle, and posterior portions of the tongue during spontaneous swallowing of saliva in each anatomic axis. In the inferior–superior axis (Fig. 3a), statistical differences in displacement were observed among all portions thus: the anterior and posterior (*p* = 0.000), anterior and middle (*p* = 0.001), and between middle and posterior portions (*p* = 0.001). The greatest displacement was observed in the posterior portion, followed by the middle and anterior portions, respectively. In the posterior–anterior axis (Fig. 3b), statistical differences in displacement were observed between anterior and middle portions (*p* = 0.002) and between anterior and posterior portions (*p* = 0.039). The anterior portion revealed the lowest movement in comparison to the other two portions. Finally, in the medial–lateral axis (Fig. 3c), there were no statistical differences in displacement among the different tongue portions.

**Table 1:** Tongue portions displacement range (mm) during spontaneous deglutition of saliva

Axis	Tongue portions		
	Anterior	Middle	Posterior
Inferior–superior	5.94 (Q1 = 3.8; Q3 = 9.5)^a,b^	9.71 (Q = 18.2; Q3 = 12.7)^a,c^	14.38 (Q1 = 11.6; Q3 = 17.5)^b,c^
Posterior–anterior	6.16 (Q1 = 3.7; Q3 = 11.6)^d,e^	11.04 (Q1 = 8.1; Q3 = 15.1)^d^	10.00 (Q1 = 8; Q3 = 12.7)^e^
Medial–lateral	3.00 (Q1 = 2; Q3 = 6.1)	3.49 (Q1 = 2.5; Q3 = 5.4)	3.93 (Q1 = 3.1; Q3 = 4.8)

The letters a–e represent significant differences among tongue portions on the same axis, in accordance with Fig. 3

### Tongue Movement During Water Deglutition

#### Different Water Volumes

Tables 2, 3, and 4 show the displacement range in millimeters of the different tongue portions while swallowing 1-AVS, ½-AVS, and ¼-AVS, according to the inferior–superior, posterior–anterior, and medial–lateral axis, respectively.

**Table 2:** Tongue portion displacement range (mm) during swallowing of different water volumes, on the inferior–superior axis

	1-AVS	½-AVS	¼-AVS
Anterior	13.5 ± 3.5^a/i,ii^	12.5 ± 4.3^iii^	10.9 ± 4^a/iv^
Middle	15.8 ± 4.3^b,c/i^	13.4 ± 3.3^b^	12.1 ± 3.7^c^
Posterior	17 ± 3.6^d,e/ii^	14.8 ± 4.2^d/iii^	13.8 ± 4.6^e/iv^

**Fig. 4:**
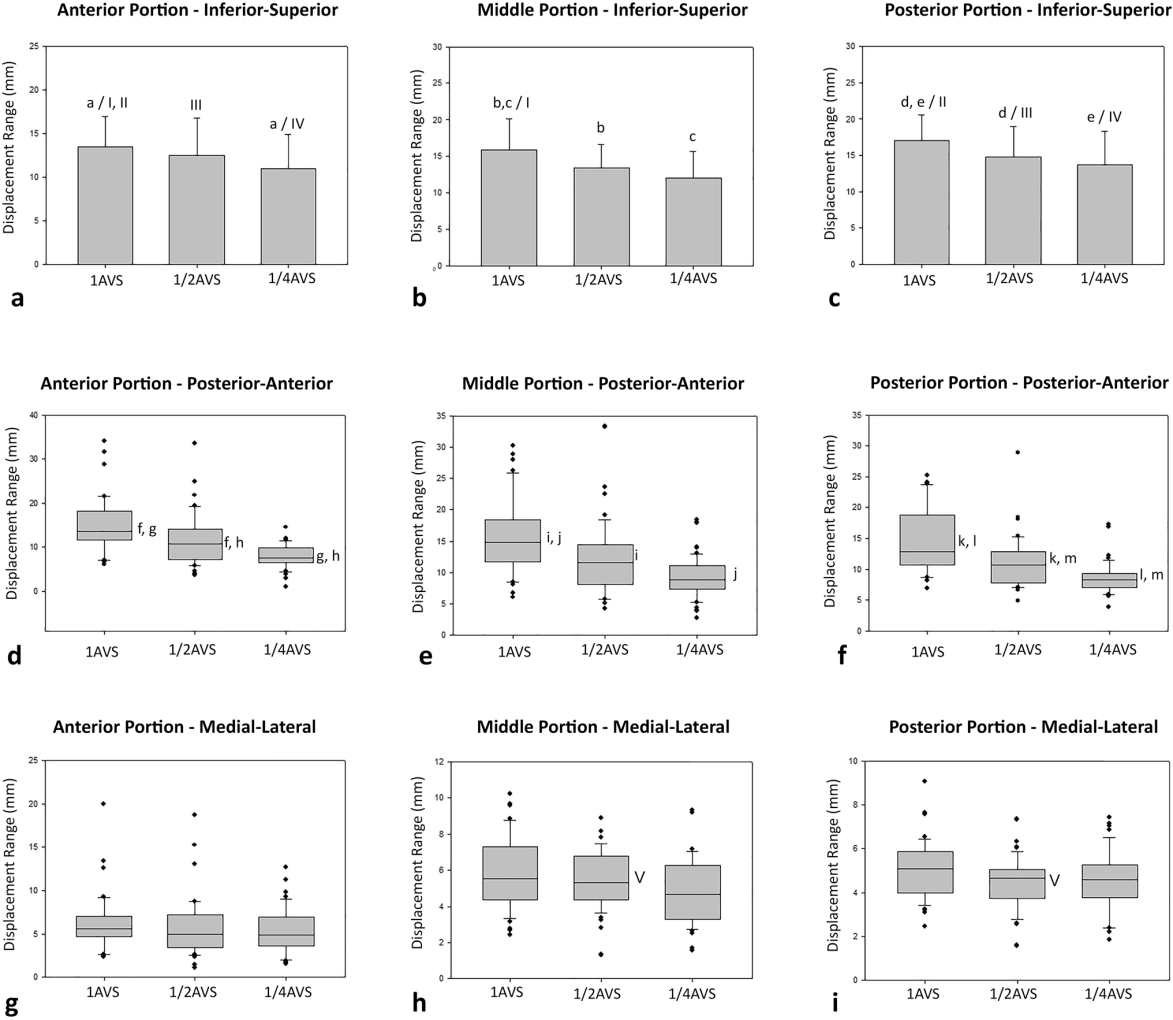
Range of displacement of the anterior (**a**, **d**, **g**), middle (**b**, **e**, **h**), and posterior (**c**, **f**, **i**) tongue portions while swallowing different water volumes on the inferior–superior (**a**–**c**), posterior–anterior (**d**–**f**), and medial–lateral (**g**–**i**) axes. The letters (a–m) indicate intra-graph significant differences in different volumes for the same tongue portion, and the characters (i–v) indicate inter-graph statistical differences among portions for the same volume

The letters a, b, c, d, and e represent significant differences among volumes in the same tongue portion. The numbers i, ii, iii, and iv represent significant differences among tongue portions in the same volume, in accordance with Fig. 4a–c

**Table 3:** Tongue portions displacement range (mm) during swallowing of different water volumes, on the posterior–anterior axis

	1-AVS	½-AVS	¼-AVS
Anterior	13.55 (Q1-11.72; Q3-18.15)^f,g^	10.82 (Q1-7.19; Q3-14.08)^f,h^	7.57 (Q1-6.55; Q3-9.91)^g,h^
Middle	14.88 (Q1-11.73; Q3-18.4)^i,j^	11.58 (Q1-8.12; Q3-14.44)^i^	8.89 (Q1-7.43; Q3-11.09)^j^
Posterior	12.86 (Q1-10.68; Q3-18.78)^k,l^	10.71 (Q1-7.8; Q3-12.81)^k,m^	8.31 (Q1-7.09; Q3-9.3)^l,m^

The letters f–m represent significant differences among volumes in the same tongue portion, in accordance with Fig. 4d–f

**Table 4:** Tongue portions displacement range (mm) during swallowing of different water volumes, on the medial–lateral axis

	1-AVS	½-AVS	¼-AVS
Anterior	5.62 (Q1-4.67; Q3-7.02)	4.99 (Q1-3.48; Q3-7.21)	4.91 (Q1-3.67; Q3-6.97)
Middle	5.54 (Q1-4.36; Q3-7.35)	5.33 (Q1-4.32; Q3-6.78)^v^	4.68 (Q1-3.26; Q3-6.31)
Posterior	5.08 (Q1-3.93; Q3-5.88)	4.65 (Q1-3.71; Q3-5.06)^v^	4.59 (Q1-3.621; Q3-5.33)

The character v represents significant differences between middle and posterior tongue portions in ½-AVS, in accordance with Fig. 4h and i

In the inferior–superior axis (Table 2), a statistical difference in displacement of the anterior tongue portion was found between swallows of 1-AVS and ¼-AVS (Fig. 4a; *p* = 0.012); for the middle tongue portion, statistical differences in displacement were found between swallows of 1-AVS and ½-AVS (Fig. 4b; *p* = 0.013) and between swallows of 1-AVS and ¼-AVS (Fig. 4b; *p* = 0.000); in the posterior tongue portion, statistical differences in displacement were found between swallows of 1-AVS and ½-AVS (Fig. 4c; *p* = 0.039) and between swallows of 1-AVS and ¼-AVS (Fig. 4c; *p* = 0.002). In all cases, the tongue displacements were greater to greater volume of water swallowed.

In the posterior–anterior axis (Table 3), statistical differences in displacement of the anterior tongue portion were observed between all evaluated volumes: 1-AVS and ½-AVS (Fig. 4d; *p* = 0.025), between 1-AVS and ¼-AVS (Fig. 4d; *p* = 0.000), and between ½-AVS and ¼-AVS (Fig. 4d; *p* = 0.003). In the middle portion, statistical differences in displacement were noted between swallows of 1-AVS and ½-AVS (Fig. 4e; *p* = 0.02) and swallows of 1-AVS and ¼-AVS (Fig. 4e; *p* = 0.000). In the posterior tongue portion, significant differences in displacement were found between swallows of 1-AVS and ½-AVS (Fig. 4f; *p* = 0.000); between swallows of 1-AVS and ¼-AVS (Fig. 4f; *p* = 0.000); and between swallows of ½-AVS and ¼-AVS (Fig. 4f; *p* = 0.033). One more time, in all cases of significant differences, the movements were broader, the larger the swallowed volume.

Finally, on the medial–lateral axis (Table 4) for the displacement of the anterior tongue portion (Fig. 4g); no statistical differences were found among the different swallowed volumes (*p* = 0.283). The middle portion (Fig. 4h) did not reveal a statistical difference among the portions (*p* = 0.05), and the posterior portion (Fig. 4i) showed no statistical differences between volumes (*p* = 0.129).

### Different Tongue Portions

Analysis of the displacement range in millimeters of the different tongue portions in the inferior–superior axis (Table 2) for swallowing 1-AVS yielded statistical differences between the movement of the anterior and middle portions (Fig. 4a, b; *p* = 0.023) and between anterior and posterior (Fig. 4a, c; *p* = 0.000). In swallowing ½-AVS and ¼-AVS, a statistical difference was observed only between the movement of the anterior and posterior tongue portions (*p* = 0.029 and *p* = 0.006, respectively; Fig. 4a, c). On this axis, in all cases, the larger movements were recorded in the posterior and middle portions.

On the posterior–anterior axis, statistical differences in the range of displacement of the different tongue portions (Table 3) for the swallows of 1-AVS (Fig. 4d; *p* = 0.730), ½-AVS (Fig. 4e; *p* = 0.717), and ¼-AVS (Fig. 4f; *p* = 0.064) were not found.

And finally, in the range of displacement of the different tongue portions in the medial–lateral axis (Table 4), statistical difference was found only for ½-AVS swallowing between the movement of the middle and posterior tongue portions (Fig. 4h, i; *p* = 0.006). Contrary to what was observed previously, the medial portion showed broader movements on the medial–lateral axis.

## Discussion

The lingual movements are biomechanically complex. The tongue is characterized as a muscular hydrostat whose operation resembles a hydraulic system, different from most of the muscles that normally behave as a mechanical system [18]. In functional activities, such as swallowing, the tongue performs movements that have been described using X-ray, ultrasound, and magnetic resonance-based measuring instruments. These devices provide information on tongue structure and position; however, their spatial and temporal resolution is limited in tasks that require high precision [19]. The previous identification of tongue movement patterns in physiological swallowing of saliva [1, 10] was performed using techniques that register the articulation of the lingual movement in two dimensions. However, we must consider that the tongue is contained within the oral cavity and that its complex displacement occurs in three axes. Thus the movements of the tongue during the physiological swallowing of saliva and voluntary swallowing of water have not been characterized three-dimensionally according to the range of displacement of tongue portions (anterior, middle, and posterior), the relation of them to the palate with high precision (0.3 mm), and temporal resolution (sample frequency of 1 kHz), as proposed in this study.

The movements for each functional task of swallowing were repeated three times, because that was considered a number of acceptable repetitions to represent a behavior [20]. The recordings showed constant patterns of swallowing in the same individual when performing three repetitions. It was possible to determine patterns of salivary swallowing previously described [9, 10]. In previous studies, salivary swallowing pattern type I, analogous to tipper, was predominant [9, 10], differing with our results that showed a high prevalence of the salivary swallowing pattern type III, a variation of type II (analogous to dipper). The disagreement of the results could be attributed to the differences between the techniques of analysis. Doods et al. [9], used videofluoroscopy, and Bourdiol et al. [10], used the 2D-electromagnetic articulography. Furthermore, in none of these cases was the tongue displacement considered in relation to an anatomic structure, such as the hard palate, as we proposed in the present study.

In our study, we could observe that performing specific swallowing tasks, the tongue did not present a symmetric movement in three dimensions but, rather, the action of intrinsic and extrinsic muscles translated into movements that differ among the tongue portions themselves and among the anatomic axes. In the three-dimensional analysis of the lingual physiological swallowing of saliva, greater differences in displacement were observed in the sagittal plane, specifically in the inferior–superior axis, and all the portions presented differences among them. The posterior portion presented greater displacement, followed by the middle and anterior portions, respectively. The movements in the posterior–anterior axis also showed differences; again, the anterior portion of the tongue presented lower displacement compared to the middle and posterior portions, which were similar to each other. The medial–lateral axis did not reveal differences in the movements of the tongue portions in the physiological swallowing of saliva in the volunteers. The posterior portion of the tongue seems to participate more actively on saliva propulsion independently of the deglutition pattern observed.

The analysis of our data showed that the inferior–superior and posterior–anterior axes evaluated in other studies [11, 21, 22] presented the greatest variations of tongue movements during the physiological swallowing of saliva. Tasko et al. [21], employing a microbeam X-ray system, also reported results of wider movements in the posterior portion and smaller movements in the anterior portion of the tongue while swallowing saliva. Steele et al. [11] performed an analysis of these movements in salivary swallowing by considering two axes of motion. In the inferior–superior axis, wider movements in the posterior portions were observed, and on the posterior–anterior axis, the anterior portion showed smaller displacements. Our results corroborate the observations of both studies; however, the values of the amplitude of movements were different.

The swallowing of different volumes of water showed variations among the portions of the tongue. In the movements of the inferior–superior axis, the posterior portion revealed the largest displacements and the anterior portion the smallest ones. In all portions, the swallowed volume reflected directly on tongue movements of this axis; thus, higher volumes resulted in greater displacements. The analysis of the movements on the posterior–anterior axis also presented displacement differences directly proportional to the swallowed volume; however, no differences were noted among the tongue portions for a same-liquid volume. This difference between the inferior–superior and posterior–anterior axes may be due to a more uniform anteroposterior tongue movement for water swallowing, not significant stretching or shortening. Finally, the medial–lateral axis showed significant difference only in the comparison of the middle and posterior portions while swallowing ½-AVS. The reduced tongue movement in the medial–lateral axis could be attributed to space limitation. The tongue was confined within dental arches as we only include participants with complete dentition. It would be interesting to observe what happens in edentulous participants.

Previous studies have analyzed muscle activity while swallowing different liquid volumes, using electromyography of the palatine veil muscles [23] and supra-hyoid region [24]. In both cases, a pattern of greater activity was observed while swallowing larger volumes of fluid. Despite differences regarding technique and regions of the tongue analyzed, the results from the present study corroborates previous findings [22–24]. Swallowing larger liquid volumes required higher functional costs to the stomatognathic system.

The comparison between movement of tongue portions while swallowing saliva and water revealed that the movements in the inferior–superior axis presented the greatest variations, and the medial–lateral axis showed almost no significant differences. The tongue portion that performed greater movements in both types of deglutition was the posterior one, and the smaller movements were registered in the anterior one. The greatest difference between the two types of deglutition was observed on the anteroposterior axis movement, in which swallowing saliva presented better-established differences between the portions of the tongue when compared to swallowing of different volumes of water.

Among the limitations of the present study, we can identify the discomfort produced by the cables that connect the sensors to the apparatus and the incorporation of water as only substance to be swallowed.

This study was the first to evaluate tongue movements during swallowing on the medial–lateral axis. Discrete difference was observed among tongue portions, volumes, and swallowed elements (saliva/water). This axis may not reveal major changes related to lingual movements during swallowing in this study. However, future studies including participants with swallowing problems may reveal the importance of the analysis of this axis that has been disregarded in the past. The literature reveals that there is an association between tongue movements during swallowing and dentomaxillary morphology [25–27], indicating that dentofacial morphology influences lingual dorsum position in swallowing, reporting a great variability in patients with class III malocclusion. Future investigations of our research group will consider participants of different dental and skeletal classifications to evaluate possible changes in lingual movements during swallowing. Finally, the improvement in comprehension of these complex functional movements can generate tri-dimensional classification patterns tongue movements during swallowing. In this stage of the study, the patterns of swallowing were successfully identified, but due to the uneven number of cases no comparison of lingual movement among patterns was made, however this will be addressed in future investigations.

The method of analysis proposed in the present study was able to distinguish the diversity of movement among tongue portions in three axes, implying that there is a joint and sequence of movement during the execution of swallowing, whose main novelties, compared to previously used methods, were the analysis of the movements associated with an anatomic structure, the palate, and the unpublished analysis of the movements on the medial–lateral axis.

## Conclusion

The movements of the tongue portions were influenced by the volume and the element to be swallowed (saliva or water). The major displacements registered in the inferior–superior and posterior–anterior axes occurred in the posterior portion and the smaller ones in the anterior portion. The amplitude of the movement was directly proportional to the volume of water swallowed, corroborating previous studies that showed similar behavior. However, the measured values were discrepant, thus demonstrating the need for more studies with adequate methods that deliver a high amount of information and high temporal and spatial precision that could clarify the swallowing tongue movements.
